# A Static Damage Constitutive Model of Concrete Based on Microscopic Damage Mechanism

**DOI:** 10.3390/ma17010117

**Published:** 2023-12-26

**Authors:** Ying Xie, Zhiwu Yu

**Affiliations:** 1 Department of Building Engineering, Hunan Institute of Engineering, Xiangtan 411104, China; 2 Hunan Provincial Key Laboratory of Intelligent Disaster Prevention-Mitigation and Ecological Restoration in Civil Engineering, Xiangtan 411104, China; 3 School of Civil Engineering, Central South University, Changsha 410075, China; zhwyu@csu.edu.cn

**Keywords:** constitutive model, random field theory, parameter identification, multi-scale modeling

## Abstract

In this article, a microscopic constitutive model is established that includes friction, plastic, and spring elements and has clear physical meaning. The friction unit reflects the mutual friction between crack surfaces, the plastic unit reflects the development of concrete plasticity, and the fracture of the spring unit reflects the formation and expansion of interior cracks in concrete. In addition, the integration of the random field theory into this model uncovers the physical underpinnings of the relationship between concrete’s nonlinearity and randomness. The multi-scale modeling of the concrete static damage constitutive model is then realized once the parameters of the random field are discovered using the macro test results. In order to apply the model’s applicability in finite element programs, a subroutine was ultimately constructed. The experimental data and the anticipated values from the numerical simulation are in good agreement, supporting the model’s realism.

## 1. Introduction

In recent decades, academics have concentrated on constitutive modeling and nonlinear analysis of concrete materials. There has been significant advancement in the aforementioned problems thanks to the efforts of researchers. However, given the complexity of concrete materials, this subject still presents many difficulties [1,2,3,4]. The primary characteristic of concrete materials is the non-uniformity of its internal structure. The concrete material’s stress–strain curve exhibits a clear nonlinear connection when it is subjected to external load [5,6,7]. The cause is the nucleation and propagation of microcracks and microdefects in concrete materials due to external loads, which causes localized stress concentration and degradation of the stress–strain curve. Therefore, due to the widespread use of continuum damage mechanics (CDM) to describe and model the issues with concrete materials over the past 20 years, numerous significant advancements have been made [8,9,10,11,12]. It can accurately realize the building of concrete material constitutive relationships since it is based on the developed irreversible thermodynamics theory system. The special properties of concrete materials, such as strain hardening, strain softening, residual stress, etc., are effectively reflected in the theoretical results based on continuum damage mechanics, and the engineering application is also quite successful. However, the majority of continuum damage mechanics-based theoretical models can only employ the empirical damage development equation to derive the damage variables, which results in the lack of parameters with a clear physical meaning.

Therefore, because of the precise physical description of their properties, fiber bundle models (FBMs) have received increased attention within the context of damage mechanics. This model was created using random properties and micromechanics of materials. It contends that throughout the creation process of concrete materials, numerous distributed microcracks and pores gradually occur. These nucleated microcracks and pores will cause the formation of random damage when subjected to load. The FBM first appeared in the paper published by Peirce in 1926 [13], and then Daniels [14] made a detailed static analysis of it. Later, some researchers also further developed the FBMs [15,16]. However, in the above research, only the elastic damage (stiffness degradation) caused by fiber fracture in concrete is considered, while the plastic damage is ignored. In 1982, Krajcinovic and Silva [17] used the joint probability density method to couple the elastic-plastic damage in concrete materials and proposed a model represented by a series of parallel micro units with the same stiffness. The elastic damage variable of the model is defined as the fracture probability of microscopic units, while plasticity is characterized by the units in the toughness model proposed by Iwan [18].

After that, researchers have also studied and developed FBMs, considering plastic damage [19,20,21,22,23,24,25]. For example, reference [19] proposed a kind of plastic fiber bundle model (PFBM) that can still bear a part of the force less than its breaking strength after the spring chain is broken. Le [20] et al. proposed a stochastic microscopic damage model and predicted the static strength and crack development of concrete materials under load. Based on the fiber chain element proposed by Li and Ren [12], Feng et al. [21] connected the plastic slider in series to characterize the hysteretic properties of concrete materials. Yu and Shan [22] introduced an irrecoverable plastic element to characterize the elastic damage and irrecoverable damage characteristics. Chen and Li [23] conducted dimensionality reduction parameter identification based on constrained optimization problems for the model proposed in reference [12]. Guo and Kuang [24] proposed a uniaxial stochastic micromechanical damage model to describe the elastoplastic response of quasi-brittle materials subjected to monotonic and cyclic loading. Voyiadjis et al. [25] developed an anisotropic elasto-plastic damage model for quasi-brittle materials within the thermodynamics framework.

However, there are some defects in the above models. The model in the literature [19] lacks clear physical meaning for the characterization of plasticity in concrete materials. In the model proposed by Feng et al. [22], the plastic slider is only used to characterize the macroscopic plastic strain and stress, and the corresponding result is based on the empirical formula, which leads to the low accuracy of the calculated result. In the model proposed by Shan and Yu [23], the damage evolution equation is determined by assuming a probability density cumulative distribution function with multiple coefficients. Due to the complexity of their establishment methods, other FBMs considering plastic damage are difficult to determine in numerical simulation. The method proposed in reference [24] requires a large number of uniaxial tension–compression complete curves of concrete with different strengths. The model proposed in reference [25] cannot represent the hysteresis loading with minor cyclic loops and is limited to one dimension.

Therefore, based on the above conclusions, a coupled microscopic model of elastic-plastic damage has been established, which can consider the plastic development of concrete and the generation, development, and interaction of internal cracks in concrete. At the same time, random field theory was introduced into the microscopic model to reveal the physical essence of the coupling between nonlinearity and randomness in concrete. This model can be used to describe the physical properties of concrete materials under uniaxial and biaxial loading and can be better used in the mechanical analysis of concrete structures.

## 2. Constitutive Model of Static Damage of Concrete

### 2.1. Proposal of Meso Physical Model

**Figure 1:**
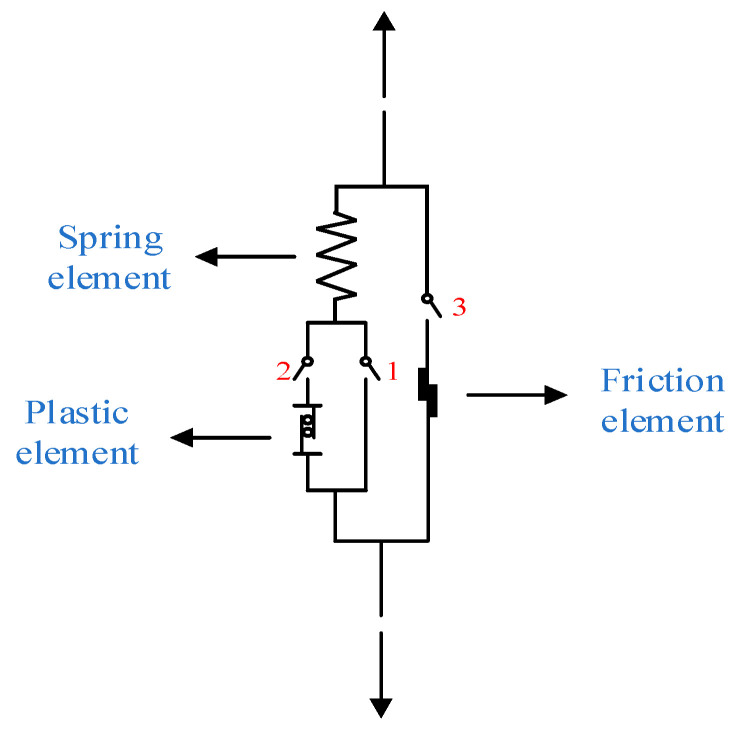
The mesophysical model.

Using damage mechanics and meso statistical mechanics, a new meso physical model is created to explain the nonlinearity and unpredictability of concrete materials. The model consists of three elements: a spring element, friction element, and plastic element, as shown in Figure 1. The spring element is compressed and deformed by an external tension when under the influence of an external load. When the spring element reaches the fracture threshold and the spring snaps, the friction element begins to bear tension and generate sliding friction. The introduction of plastic components is a sign of the expansion of material plasticity. As the spring element draws closer to the yield threshold, the plasticity starts to slide. In addition, this work also introduces the random field theory to reflect the unpredictable nature of microfracture and element production. It is assumed that the yield threshold of each plastic element and the fracture threshold of each spring element both follow a random distribution. In other words, the way a micro-spring quits working and the way plastic yields are both random events.

The model has three switches that regulate the operation of various elements. When switch 1 is closed and switches 2 and 3 are open, only the spring element operates in the entire model to represent the linear elastic component of concrete. When switch 2 is closed and switches 1 and 3 are open, the series element made up of a spring element and a plastic element acts in the model. At this time, the plastic element slides, which indicates the plastic development of concrete materials. When switch 3 is closed and switches 1 and 2 are opened, only the friction element acts in the whole model to express the residual stress caused by the friction between crack surfaces after concrete cracking. So, when the concrete material is in the ideal initial lossless state, the model is in the state of switch 1 closed and switch 2 and 3 opened. Then, with the increase in external load, switches 2 and 3 are triggered, respectively. In the whole force process, only one of the three switches of the model is closed, and the other two are opened.

It is assumed that the threshold of spring fracture in the model is $\Delta_{r}$, and the yield threshold of plastic sliding chain is $\Delta_{y}$. When element strain $\varepsilon \leq \Delta_{r},\varepsilon \leq \Delta_{y}$, the spring element does not break, and the plastic element does not yield. At this time, only the spring element is in the working state, that is, switch 1 is closed and switches 2 and 3 are opened. So, the control function of switch 1 can be expressed as

(1) $$S1=H(\Delta_{r}-\varepsilon )H(\Delta_{y}-\varepsilon )$$

where H(⋅) denotes Heaviside function:

(2) $$H(x)=\left\{\begin{array}{l} 0\;\;\;\;(x\leq 0)\\ 1\;\;\;\;(x>0)\;\; \end{array}\right.$$

So, the Formula (1) can be expressed as

(3) $$S1=H(\Delta_{r}-\varepsilon )H(\Delta_{y}-\varepsilon )=\left\{\begin{array}{l} 1\;\;\;\;\;switch\;on\\ 0\;\;\;\;\;\;switch\;off \end{array}\right.$$

When $\varepsilon \geq \Delta_{y},\varepsilon \leq \Delta_{r}$, the spring element does not break, and the plastic element begins to yield. At this time, the series element composed of spring element and plastic element in the model acts, that is, switch 2 is closed, and switches 1 and 3 are opened. So, the control function of switch 2 can be expressed as

(4) $$S2=H(\varepsilon -\Delta_{y})H(\Delta_{r}-\varepsilon )=\left\{\begin{array}{l} 1\;\;\;\;\;switch\;on\\ 0\;\;\;\;\;\;switch\;off \end{array}\right.$$

When $\varepsilon \geq \Delta_{r},\varepsilon \leq \Delta_{y}$, the spring element is broken, and the friction element is sliding. At this time, only the friction element in the model is in the working state, that is, switch 3 is closed and switches 1 and 2 are opened. So, the control function of switch 3 can be expressed as

(5) $$S3=H(\varepsilon -\Delta_{r})H(\Delta_{y}-\varepsilon )=\left\{\begin{array}{l} 1\;\;\;\;\;switch\;on\\ 0\;\;\;\;\;\;switch\;off \end{array}\right.$$

### 2.2. Mechanical Characteristics of Single Element

According to the above definition, the stress–strain relationship of each element in the model can be expressed as follows:

(1) The spring element:

(6) $$\sigma_{e}=H(\Delta_{r}-\varepsilon )H(\Delta_{y}-\varepsilon )E\varepsilon$$

where *E* denotes the elastic modulus.

(2) The plastic element

It has been pointed out in the literature [26] that the bearing capacity of concrete material will gradually decrease with the increase in plastic strain after crossing the peak point of the stress–strain curve. The law can be expressed as

(7) $$\frac{d\sigma }{du_{p}}=-K$$

where $u_{p}$ denotes the plastic deformation, and *K* is a constant. The relationship between $u_{p}$ and plastic strain can be expressed as

(8) $$\varepsilon_{p}=\frac{u_{p}}{L}$$

where L denotes the length of the specimen. So, the Formula (7) can be expressed as

(9) $$\frac{d\sigma }{d\varepsilon_{p}}=-KL=-Y$$

**Figure 2:**
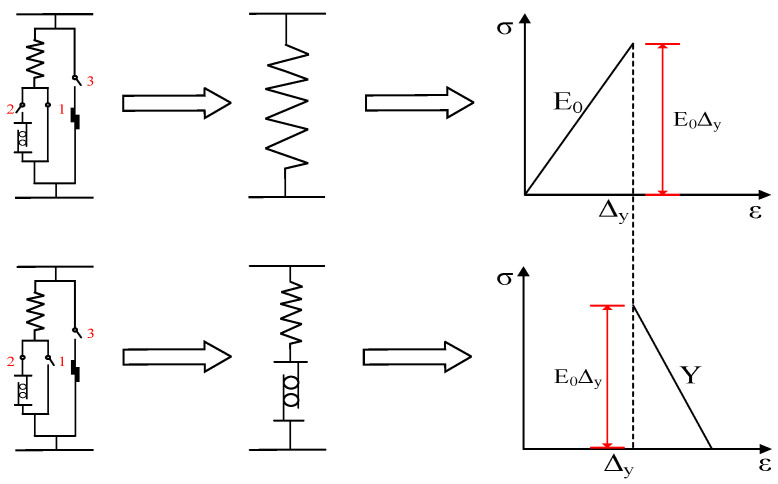
Stress characteristics of plastic elements.

In order to describe the fact that the bearing capacity of concrete gradually decreases with the development of plasticity, the model assumes that when $\;\varepsilon \geq \Delta_{y}$, the spring element shrinks with the sliding of plasticity. At this time, the stress of the series element composed of the spring element and plastic element decreases with the increase in strain. For simplicity, it is assumed that the model stress decreases linearly with the increase in strain, and the stress characteristics of the element are shown in Figure 2.

Therefore, the stress–strain relationship of the plastic element can be expressed as

(10) $$\sigma_{p}=[E\Delta_{y}+Y(\varepsilon -\Delta_{y})]H[E\Delta_{y}+Y(\varepsilon -\Delta_{y})]H(\varepsilon -\Delta_{y})H(\Delta_{r}-\varepsilon )$$

(3) The friction element

According to the literature [27], the relationship between the residual stress $\sigma_{r}$ and the fracture stress $\sigma_{c}$ of concrete can be expressed as follows:

(11) $$\sigma_{r}=\eta_{s}\sigma_{c}$$

where $\eta_{s}$ denotes the shear lag coefficient. So, from Equations (6) and (11), it can be concluded that

(12) $$\sigma_{r}=\eta_{s}\sigma_{c}=\eta_{s}E\Delta_{r}H(\varepsilon -\Delta_{r})H(\Delta_{y}-\varepsilon )$$

**Figure 3:**
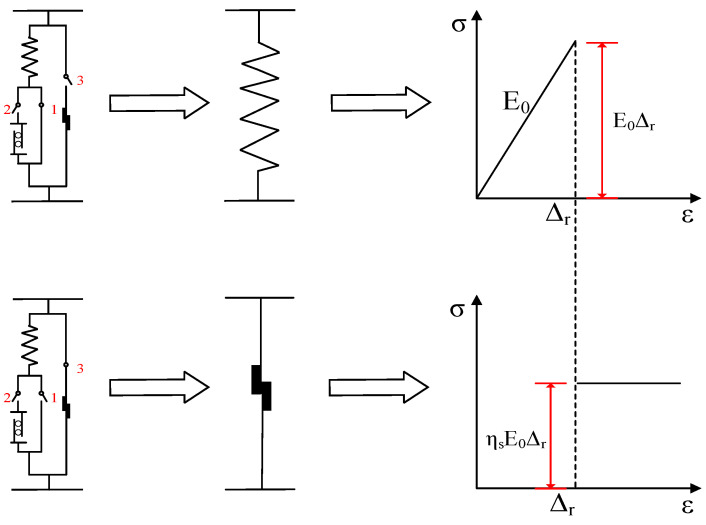
Stress characteristics of friction block element.

Therefore, the stress characteristics of the friction block element can be shown in Figure 3.

For the sake of simplification, the following assumptions can be made:

(13) $$\begin{array}{l} \varphi_{1}=H\left(\Delta_{r}-\varepsilon \right)H\left(\Delta_{y}-\varepsilon \right)\\ \varphi_{2}=H\left(\Delta_{r}-\varepsilon \right)H\left(\varepsilon -\Delta_{y}\right)H\left[E{∆}_{y}+Y\left(\varepsilon -\varepsilon_{y}\right)\right]\\ \varphi_{3}=H(\varepsilon -\Delta_{r})H(\Delta_{y}-\varepsilon ) \end{array}$$

Therefore, the total stress–strain relationship of a single meso physical model can be expressed as

(14) $$\sigma =\varphi_{1}E\varepsilon +\varphi_{2}[E\Delta_{y}+Y(\varepsilon -\Delta_{y})]+\varphi_{3}\eta_{s}E\Delta_{r}$$

### 2.3. Parallel Bar System

**Figure 4:**
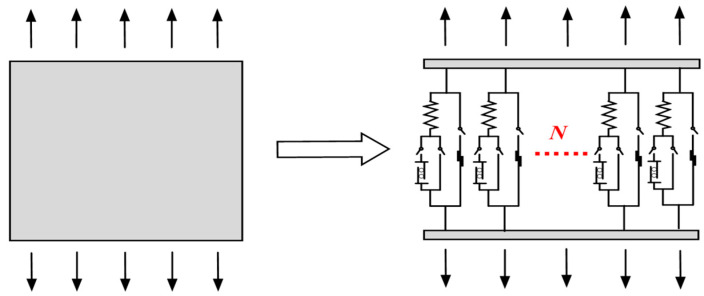
Schematic diagram of parallel system.

On the basis of the previous section, *N* meso units are combined into a parallel system to simulate the stress of concrete, as shown in Figure 4.

Therefore, the stress of the *k*th meso element $\sigma^{k}$ can be expressed as

(15) $$\sigma^{k}=\sigma (x_{k}),k=1,2,3\ldots \ldots N$$

where $x_{k}$ denotes the space coordinates of the *k*th meso element, and *N* denotes the total number of meso units.

Assuming that each meso element has the same section form, the stress of *N* parallel meso elements can be expressed as

(16) $$\sigma_{e}=\frac{1}{N}\sum_{k=1}^{N}H[\Delta_{r}(x_{k})-\varepsilon ]H[\Delta_{y}(x_{k})-\varepsilon ]E\varepsilon$$

(17) $$\sigma_{p}=\frac{1}{N}\sum_{k=1}^{N}\left\{\begin{array}{l} \left[E\Delta_{y}(x_{k})+Y\varepsilon -Y\Delta_{y}(x_{k})]\times H[E\Delta_{y}(x_{k})+Y\varepsilon -Y\Delta_{y}(x_{k})\right]\\ \times H[\varepsilon -\Delta_{y}(x_{k})]\times H[\Delta_{r}(x_{k})-\varepsilon ] \end{array}\right\}$$

(18) $$\sigma_{r}=\frac{1}{N}\sum_{k=1}^{N}\eta_{s}E\Delta_{r}(x_{k})H[\varepsilon -\Delta_{r}(x_{k})]H[\Delta_{y}(x_{k})-\varepsilon ]$$

When N→∞, the above formula can be expressed as follows:

(19) $$\sigma_{e}=\int_{0}^{1}H(\Delta_{r}-\varepsilon )H(\Delta_{y}-\varepsilon )E\varepsilon dx$$

(20) $$\sigma_{p}=\int_{0}^{1}[E\Delta_{y}+Y(\varepsilon -\Delta_{y})]H[E\Delta_{y}+Y(\varepsilon -\Delta_{y})]H(\varepsilon -\Delta_{y})H(\Delta_{r}-\varepsilon )dx$$

(21) $$\sigma_{r}=\int_{0}^{1}\eta_{s}E\Delta_{r}H(\varepsilon -\Delta_{r})H(\Delta_{y}-\varepsilon )dx$$

**Figure 5:**
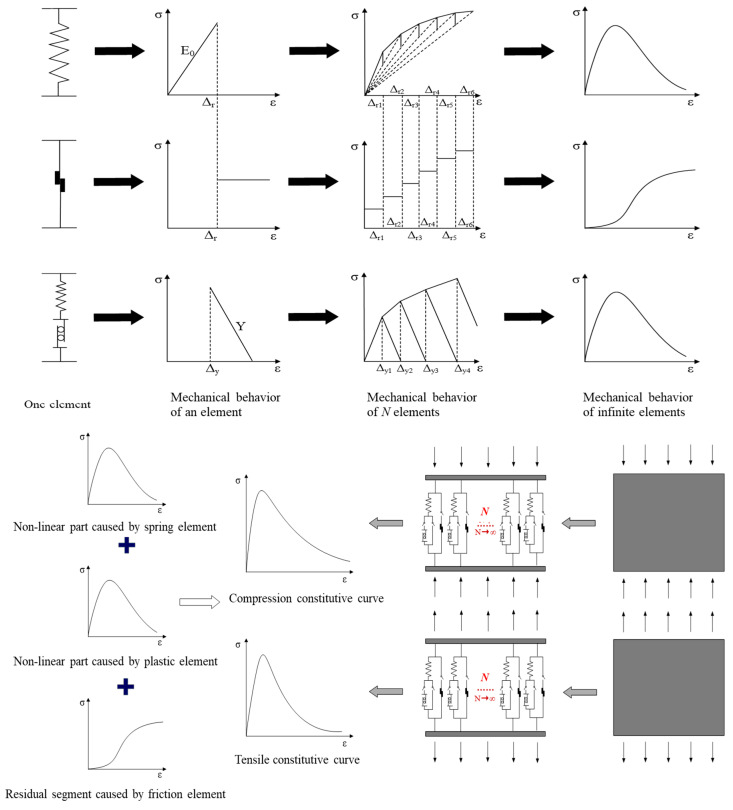
Schematic diagram of stress characteristics of a unit-discrete system-continuous system.

From a single meso element to a discrete system with *N* meso elements, and then to a continuous system with infinite meso elements, the mechanical characteristics of the whole process model are shown in Figure 5.

**Figure 6:**
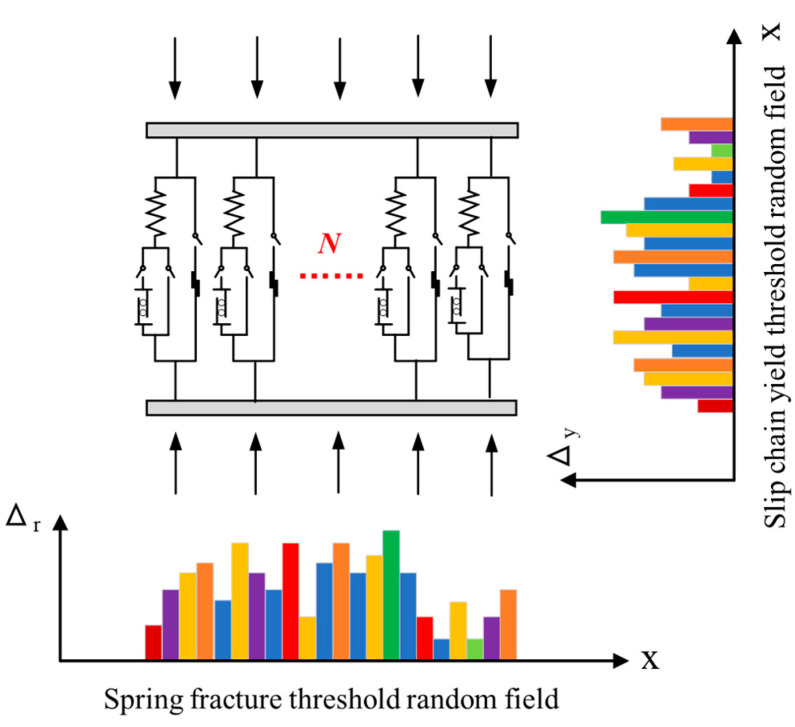
Schematic diagram of fracture threshold and yield threshold random fields.

In addition, the concept of randomness is introduced to reflect the uncontrollable nature of micro-fracture and yield. In this model, it is considered that the fracture threshold $\Delta_{r}$ of spring element satisfies $0\leq \Delta_{r}\leq \Delta_{r}^{\max}$, which probability density function is $f_{r}\left(\Delta_{r}\right)$ and distribution function is $F_{r}\left(\Delta_{r}\right)=\int_{0}^{+\infty }f_{r}(\Delta_{r})d\Delta_{r}$. It is considered that the yield threshold $\Delta_{y}$ of plastic element satisfies $0\leq \Delta_{y}\leq \Delta_{y}^{\max}$, which probability density function is $f_{y}\left(\Delta_{y}\right)$ and distribution function is $F_{y}\left(\Delta_{y}\right)=\int_{0}^{+\infty }f_{y}(\Delta_{y})d\Delta_{y}$. Additionally, $f\left(\Delta_{r},\Delta_{y},\gamma \right)$ is the two-dimensional joint probability density function of $\Delta_{r}$ and $\Delta_{y}$. $f_{r}\left(\Delta_{r}\right)$ and $f_{y}\left(\Delta_{y}\right)$ are two independent probability distributions, as shown in Figure 6.

The expected values of $\sigma_{e}$ are calculated as follows:

(22) $$\mu (\sigma_{e})=\mu [\int_{0}^{1}H(\Delta_{r}-\varepsilon )H(\Delta_{y}-\varepsilon )E\varepsilon d\varepsilon ]\;=\int_{0}^{1}\mu [H(\Delta_{r}-\varepsilon )H(\Delta_{y}-\varepsilon )E\varepsilon ]d\varepsilon$$

From the commutativity of the expectation operator and the integral operator, it can be seen that

(23) $$\begin{array}{l} \mu \left[H\left(\Delta_{r}-\varepsilon \right)H\left(\Delta_{y}-\varepsilon \right)E\varepsilon \right]\\ =1\times P\left(\varphi_{1}=1\right)\times E\varepsilon +0\times P\left(\varphi_{1}=0\right)\times E\varepsilon \\ =P\left(\varphi_{1}=1\right)\times E\varepsilon \\ =P\left(\varepsilon \leq \Delta_{r}\cap \varepsilon \leq \Delta_{y}\right)\times E\varepsilon \\ =E\varepsilon \int_{\varepsilon }^{+\infty }\int_{\varepsilon }^{+\infty }\;f(\Delta_{r},\Delta_{y};\gamma )\;d\Delta_{r}d\Delta_{y} \end{array}$$

It can be assumed that

(24) $$H_{1}=\int_{\varepsilon }^{+\infty }\int_{\varepsilon }^{+\infty }\;f(\Delta_{r},\Delta_{y};\gamma )\;d\Delta_{r}d\Delta_{y}$$

So,

(25) $$\mu (\sigma_{e})=\int_{0}^{1}\left[\int_{\varepsilon }^{+\infty }\int_{\varepsilon }^{+\infty }\;f(\Delta_{r},\Delta_{y};\gamma )E\varepsilon \;d\Delta_{r}d\Delta_{y}\right]\;dx=H_{1}E\varepsilon$$

Similarly, the expected values of $\sigma_{r}$ and $\sigma_{y}$ can be expressed as

(26) $$\begin{array}{ll} \mu (\sigma_{r}) & =\mu [\int_{0}^{1}\eta_{s}E\Delta_{r}H(\varepsilon -\Delta_{r})H(\Delta_{y}-\varepsilon )dx]\\ & =\int_{0}^{1}\mu [\eta_{s}E\Delta_{r}H(\varepsilon -\Delta_{r})H(\Delta_{y}-\varepsilon )]dx\\ & =\eta_{s}E\int_{0}^{1}\left[\int_{\varepsilon }^{+\infty }\int_{0}^{\varepsilon }\Delta_{r}f(\Delta_{r},\Delta_{y};\gamma )\;d\Delta_{r}d\Delta_{y}\right]\;dx \end{array}$$

It can be assumed that

(27) $$H_{2}\;=\int_{\varepsilon }^{+\infty }\int_{0}^{\varepsilon }\Delta_{r}f(\Delta_{r},\Delta_{y};\gamma )\;d\Delta_{r}d\Delta_{y}$$

So,

(28) $$\mu (\sigma_{r})=\eta_{s}E\int_{0}^{1}\;H_{2}dx=\eta_{s}EH_{2}$$

(29) $$\begin{array}{ll} \mu (\sigma_{p}) & =\mu \left\{\;\int_{0}^{1}\left[E\Delta_{y}+Y(\varepsilon -\Delta_{y})\right]H\left[E\Delta_{y}+Y(\varepsilon -\Delta_{y})\right]H(\varepsilon -\Delta_{y})H(\Delta_{r}-\varepsilon )\;\right\}\;dx\\ & =\int_{0}^{1}\mu \left\{\;\left[E\Delta_{y}+Y(\varepsilon -\Delta_{y})\right]H\left[E\Delta_{y}+Y(\varepsilon -\Delta_{y})\right]H(\varepsilon -\Delta_{y})H(\Delta_{r}-\varepsilon )\;\right\}\;dx\\ & =\int_{0}^{1}\left\{\;\int_{\varepsilon }^{+\infty }\int_{0}^{\varepsilon }\left[E\Delta_{y}+Y(\varepsilon -\Delta_{y})\right]H\left[E\Delta_{y}+Y(\varepsilon -\Delta_{y})\right]\;f(\Delta_{r},\Delta_{y};\gamma )\;d\Delta_{y}d\Delta_{r}\right\}\;dx \end{array}$$

It can be assumed that

(30) $$H_{3}=\int_{\varepsilon }^{+\infty }\int_{0}^{\varepsilon }\left[\Delta_{y}+Y(\varepsilon -\Delta_{y})/E\right]H\left[\Delta_{y}+Y(\varepsilon -\Delta_{y})/E\right]\;f(\Delta_{r},\Delta_{y};\gamma )\;d\Delta_{y}d\Delta_{r}$$

Then, Equation (21) can be simplified as follows:

(31) $$\mu (\sigma_{p})\;=E\int_{0}^{1}\;H_{3}\;dx=H_{3}E$$

Then, the expected value of total stress σ can be expressed as follows:

(32) $$\begin{array}{ll} \mu (\sigma ) & =\mu (\sigma_{e})+\mu (\sigma_{p})+\mu (\sigma_{r})\\ & =H_{1}E\varepsilon +H_{2}E\eta_{s}+H_{3}E \end{array}$$

### 2.4. Damage Definition

Each meso element in this parallel meso model has the same spring stiffness, and the plastic yield and fracture thresholds of the spring are random distribution field functions. Concrete is considered to have experienced elastic damage when a spring element fractures. Concrete is thought to have suffered plastic degradation from plastic elements sliding. Then, the total damage of concrete is defined as the ratio of the damaged area to the total area, which can be expressed as follows:

(33) $$d=\frac{A_{d}}{A}$$

(34) $$d_{e}(\varepsilon )=\frac{1}{N}\sum_{k=1}^{N}H[\varepsilon -\Delta_{r}(x_{k})]\cdot H[\Delta_{y}(x_{k})-\varepsilon ]$$

(35) $$d_{p}(\varepsilon )=\frac{1}{N}\sum_{k=1}^{N}H[\varepsilon -\Delta_{y}(x_{k})]\cdot H[\Delta_{r}(x_{k})-\varepsilon ]$$

When N→∞, the above formulas can be expressed as

(36) $$d_{e}(\varepsilon )=\int_{0}^{1}H[\varepsilon -\Delta_{r}(x)]\cdot H[\Delta_{y}(x)-\varepsilon ]\;dx$$

(37) $$d_{p}(\varepsilon )=\int_{0}^{1}H[\varepsilon -\Delta_{y}(x)]\cdot H[\Delta_{r}(x)-\varepsilon ]\;dx$$

(38) $$d(\varepsilon )=d_{e}(\varepsilon )+d_{p}(\varepsilon )$$

After considering the damage, the constitutive model can be expressed as

(39) $$\sigma =(1-d)E\varepsilon +\int_{0}^{1}\varphi_{2}[E\Delta_{y}+Y(\varepsilon -\Delta_{y})]\;dx+\int_{0}^{1}\varphi_{3}\eta_{s}E\Delta_{r}dx$$

The mean values of damage variables $d_{e}$, $d_{p}$ and d can be calculated as follows, respectively:

(40) $$\begin{array}{ll} \mu (d_{e}) & =\mu \left\{\int_{0}^{1}H[\varepsilon -\Delta_{r}(x)]\cdot H[\Delta_{y}(x)-\varepsilon ]\;dx\right\}\\ & =\int_{\varepsilon }^{+\infty }\int_{0}^{\varepsilon }f(\Delta_{r},\Delta_{y};\gamma )\;d\Delta_{r}d\Delta_{y} \end{array}$$

(41) $$\begin{array}{ll} \mu (d_{p}) & =\mu \left\{\int_{0}^{1}H[\varepsilon -\Delta_{y}(x)]\cdot H[\Delta_{r}(x)-\varepsilon ]\;dx\right\}\\ & =\int_{\varepsilon }^{+\infty }\int_{0}^{\varepsilon }f(\Delta_{r},\Delta_{y};\gamma )\;d\Delta_{y}d\Delta_{r} \end{array}$$

(42) $$\begin{array}{ll} \mu (d) & =\mu \left\{\int_{0}^{1}H[\Delta_{y}(x)-\varepsilon ]\cdot H[\Delta_{r}(x)-\varepsilon ]\;dx\right\}\\ & =1-\int_{\varepsilon }^{+\infty }\int_{\varepsilon }^{+\infty }f(\Delta_{r},\Delta_{y};\gamma )\;d\Delta_{r}d\Delta_{y} \end{array}$$

Therefore, μ(σ) can be expressed as follows:

(43) $$\mu (\sigma )=[1-\mu (d)]E\varepsilon +H_{2}E\eta_{s}+H_{3}E$$

When μ(d)=0, μ(σ)=Eε, which means it is in a non-destructive state. When μ(d)=1, the stress of all plastic elements in the model is 0, and $\mu (\sigma )=H_{3}E$, which means it is in a completely damaged state.

### 2.5. Biaxial Static Damage Constitutive Model of Concrete

Under the condition of two-dimensional plane stress ($\sigma_{3}=0$), the full variable form of the damage constitutive model of concrete can be expressed as follows:

(44) $$\mu (\sigma )=[I-\mu (d)]:E:\varepsilon +\eta_{s}H_{2}(\varepsilon ):E+H_{3}(\varepsilon ):E$$

where ***E*** denotes the initial material stiffness tensor of concrete, which can be expressed as follows:

(45) $$\left[E\right]=\frac{E}{1-{\nu_{0}}^{2}}\left[\begin{array}{ccc} 1 & \nu_{0} & 0\\ \nu_{0} & 1 & 0\\ 0 & 0 & (1-{\nu_{0}}^{2})G/E \end{array}\right]$$

where $\nu_{0}$ and G denotes Poisson’s ratio and the initial shear modulus of concrete.

Then, for different stress states, the damage constitutive relation of concrete can be expressed as follows:

(1) Biaxial tensile stress state ($\sigma_{1}>0,\sigma_{2}>0$):
  (46) $$\mu (\sigma )=[I-\mu (d^{+})]:E:\varepsilon +\eta_{s}H_{2}^{+}(\varepsilon ):E+H_{3}^{+}(\varepsilon ):E$$
(2) Biaxial compression stress state ($\sigma_{1}<0,\sigma_{2}<0$):
  (47) $$\mu (\sigma )=[I-\mu (d^{-})]:E:\varepsilon +\eta_{s}H_{2}^{-}(\varepsilon ):E+H_{3}^{-}(\varepsilon ):E$$
(3) Biaxial tension and compression stress state ($\sigma_{1}>0,\sigma_{2}<0$ or $\sigma_{1}<0,\sigma_{2}>0$):
  (48) $$\mu (\sigma )=\sqrt{\left[I-\mu (d^{+})]:[I-\mu (d^{-})\right]}:E:\varepsilon +\eta_{s}\sqrt{H_{2}^{+}(\varepsilon ):H_{2}^{-}(\varepsilon )}:E+\sqrt{H_{3}^{+}(\varepsilon ):H_{3}^{-}(\varepsilon )}:E$$

## 3. Identification of Random Field Parameters Based on Global Genetic Algorithm

Now, the constitutive curve of concrete is established by the method of meso random damage of concrete. The nonlinear development process of concrete macro specimens involves a complex process of loading–random damage–stress redistribution–damage evolution, as can be shown from the examination of the meso physical mechanism. Therefore, in order to model the link between meso stress and strain, the concept of multi-scale analysis is developed. This system has three fundamental scales: the micro-, meso-, and macro-scales. The stress–strain connection is determined at the micro-scale by random fracture and yield processes. Then, utilizing meso element modeling, the macroscopic mean stress–strain relationship is provided. System identification (inverse problem solving) can be used to identify the parameters of micro-fracture strain and yield strain random field from the test data of macro specimens, and the probability description of meso damage variables can then be provided. As a result, the probability description of the constitutive relation at the meso level is provided.

Using the modeling method based on the test sample set, the algorithm is divided into two processes:

First, the data of the uniaxial test of a batch of concrete specimens are interval discretized with respect to strain $\varepsilon_{i}$, and the average stress value $\left(\varepsilon_{i};\mu (\sigma_{i}^{o})\right)$ of the sample set at the corresponding point is obtained based on the midpoint of the interval. 

Second, the random field parameters of micro-fracture strain and micro yield strain are assumed. Then, the above theoretical model is used to calculate the mean stress $\mu (\sigma_{i})$ when the strain is $\varepsilon_{i}$. Finally, the unknown parameters of the random field are obtained by inverse solution with the minimum square sum of the difference between the corresponding theoretical value and the experimental value as the optimization criterion, which can be shown as

(49) $$\sum_{i=1}^{N}[\mu (\sigma_{i})-\mu (\sigma_{i}^{o}){]}^{2}\to min$$

**Figure 7:**
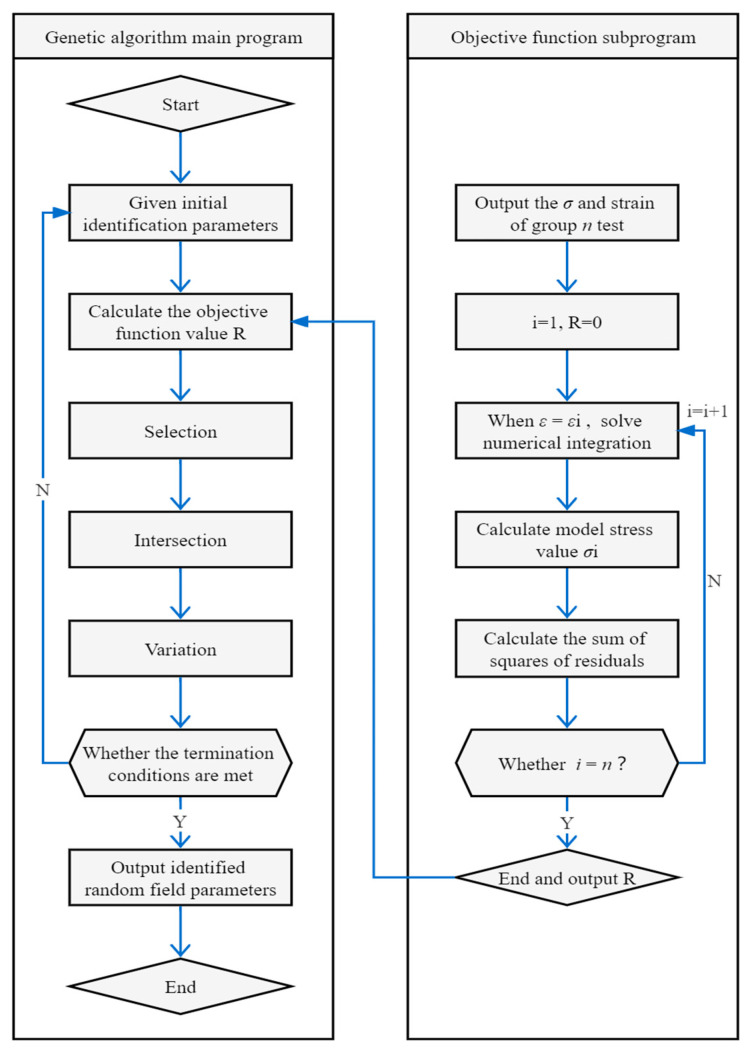
Flow chart of random field parameter identification algorithm.

Because there are many random field parameters to be identified in the model and the calculation is complex, in order to obtain an ideal optimization solution, the global genetic algorithm is used to identify the random field parameters. The calculation flow of random field parameter identification is shown in Figure 7.

## 4. Model Validation

It is assumed that the fracture threshold $\Delta_{r}(x)$ of the spring element and the yield threshold $\Delta_{y}(x)$ of the plastic element obey the lognormal distribution. Then, $Z_{r}(x)=\ln\Delta_{r}(x)$ and $Z_{y}(x)=\ln\Delta_{y}(x)$ obey normal distribution, which can be shown as $Z_{r}(x):N(\mu_{r},\sigma_{r})$ and $Z_{y}(x):N(\mu_{y},\sigma_{y})$.

Then, standardize $Z_{r}(x)$ and $Z_{y}(x)$, respectively. Let $\alpha =\left(\ln\Delta_{r}-\lambda_{r}\right)/\zeta_{r}$ and $\beta =\left(\ln\Delta_{y}-\lambda_{y}\right)/\zeta_{y}$, so α and β all follow the standard normal distribution, which can be shown as Z(α):N(0,1) and Z(β):N(0,1). So, the distribution functions and probability density functions of $\Delta_{r}(x)$ and $\Delta_{y}(x)$ ca n be shown as follows:

(50) $$F_{r}=\varphi (\frac{\ln\Delta_{r}-\lambda_{r}}{\zeta_{r}})=\frac{1}{\sqrt{2\pi }}\int_{-\infty }^{\frac{\ln\Delta_{r}-\lambda_{r}}{\zeta_{r}}}e^{-t^{2}/2}\;dt$$

(51) $$F_{y}=\varphi (\frac{\ln\Delta_{y}-\lambda_{y}}{\zeta_{y}})=\frac{1}{\sqrt{2\pi }}\int_{-\infty }^{\frac{\ln\Delta_{y}-\lambda_{y}}{\zeta_{y}}}e^{-t^{2}/2}\;dt$$

(52) $$f_{r}(\Delta_{r})=F_{r}^{\prime }=\frac{1}{\sqrt{2\pi }\Delta_{r}\zeta_{r}}\exp\left[-1/2(\frac{\ln\Delta_{r}-\lambda_{r}}{\zeta_{r}}{)}^{2}\right]$$

(53) $$f_{y}(\Delta_{y})=F_{y}^{\prime }=\frac{1}{\sqrt{2\pi }\Delta_{y}\zeta_{y}}\exp\left[-1/2(\frac{\ln\Delta_{y}-\lambda_{y}}{\zeta_{y}}{)}^{2}\right]$$

**Figure 8:**
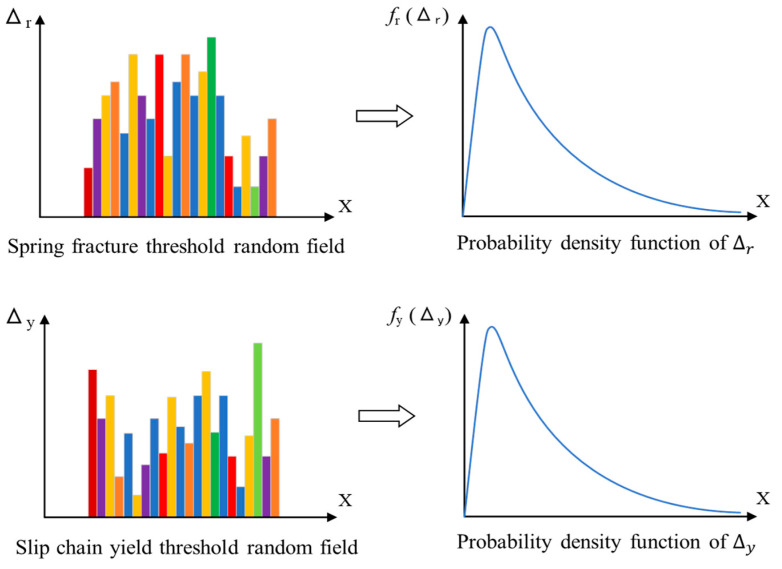
Random field distribution diagram of $\Delta_{r}(x)$ and $\Delta_{y}(x)$.

According to the above, $\Delta_{r}(x)$ and $\Delta_{y}(x)$, respectively, obey one-dimensional lognormal distribution (as shown in Figure 8), and $f(\Delta_{r},\Delta_{y};\gamma )$ is a two-dimensional lognormal distribution function. When $\Delta_{r}(x)$ and $\Delta_{y}(x)$ are independent of each other, it can be determined that

(54) $$f(\Delta_{r},\Delta_{y};\gamma )\;=f_{r}(\Delta_{r})f_{y}(\Delta_{y})$$

Then, μ(d) can be simplified as follows:

(55) $$\begin{array}{l} \mu (d)=1-\int_{\varepsilon }^{+\infty }\int_{\varepsilon }^{+\infty }f(\Delta_{r},\Delta_{y};\gamma )\;d\Delta_{r}d\Delta_{y}\\ =1-\int_{\varepsilon }^{+\infty }\int_{\varepsilon }^{+\infty }f_{r}(\Delta_{r})\;f_{y}(\Delta_{y})\;d\Delta_{r}d\Delta_{y}\\ =1-\int_{\varepsilon }^{+\infty }\int_{\varepsilon }^{+\infty }\frac{1}{2\pi \Delta_{r}\Delta_{y}}\exp\left\{-\frac{1}{2}\left[(\frac{\ln\Delta_{r}-\lambda_{r}}{\zeta_{r}}{)}^{2}+(\frac{\ln\Delta_{y}-\lambda_{y}}{\zeta_{y}}{)}^{2}\right]\right\}\;d\Delta_{r}d\Delta_{y}\\ =1-\int_{\varepsilon }^{+\infty }\left\{\int_{\varepsilon }^{+\infty }\frac{1}{\sqrt{2\pi }\Delta_{r}}\exp\;\left[-\frac{1}{2}(\frac{\ln\Delta_{r}-\lambda_{r}}{\zeta_{r}}{)}^{2}\right]\;d\Delta_{r}\right\}\frac{1}{\sqrt{2\pi }\Delta_{y}}\exp\;\left[-\frac{1}{2}(\frac{\ln\Delta_{y}-\lambda_{y}}{\zeta_{y}}{)}^{2}\right]\;d\Delta_{y} \end{array}$$

### 4.1. Concrete Uniaxial Tension and Compression

**Figure 9:**
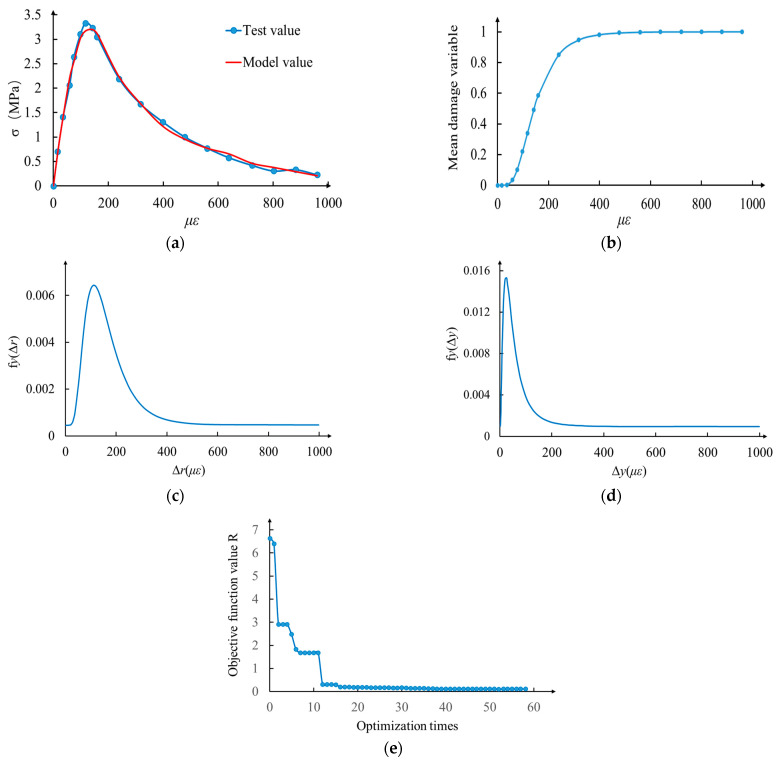
Parameter identification results of mean tension constitutive random field. (**a**) Comparison of mean tensile constitutive model values with experimental values; (**b**) change in mean tensile damage variable; (**c**) random field distribution of spring fracture threshold; (**d**) random field distribution of plastic yield threshold; (**e**) the objective function changes with optimization algebra.

**Figure 10:**
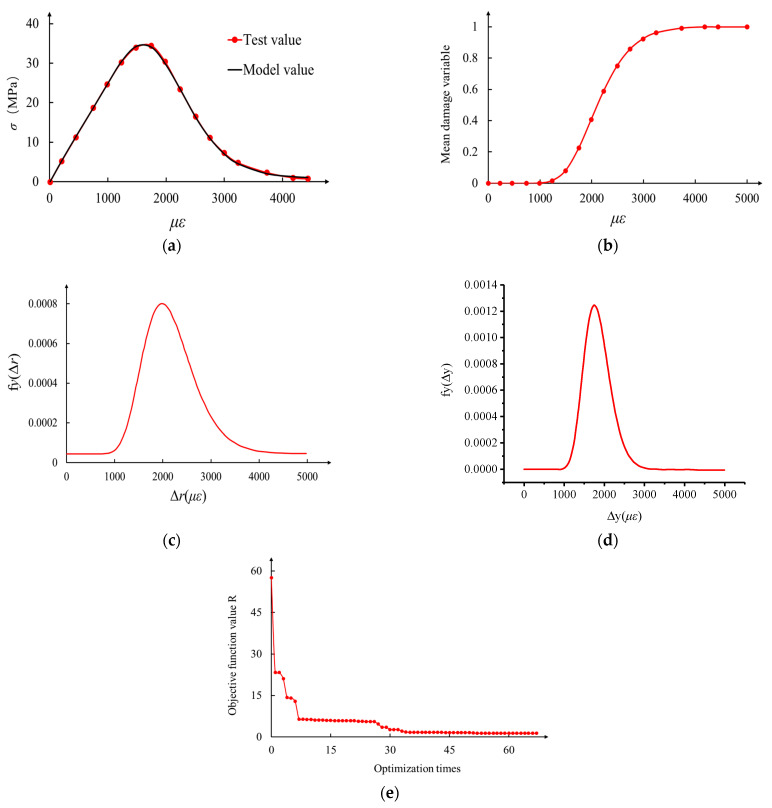
Parameter identification results of mean compressive constitutive random field. (**a**) Comparison of mean compressive constitutive model values with experimental values; (**b**) change in mean compressive damage variable; (**c**) random field distribution of spring fracture threshold; (**d**) random field distribution of plastic yield threshold; (**e**) the objective function changes with optimization algebra.

In this paper, the random field parameter identification is carried out by using the uniaxial compression and tension test data of self-compacting concrete in reference [28]. The elastic modulus of concrete measured in the test is $E_{0}=23.22\;\mathrm{GPa}$. The parameters to be identified in the model are $\lambda_{r},\;\;\zeta_{r},\;\;\lambda_{y},\;\;\zeta_{y},\;\;Y,\;\;\eta_{s}$. According to the algorithm proposed in Section 3, the parameter identification results are obtained, as shown in Figure 9 and Figure 10 and Table 1.

From the above recognition results, it can be seen that the R-value of the objective function of the genetic algorithm is very small, close to 0. Therefore, the model proposed in this paper is in good agreement with the test results, so the rationality of this model can be verified.

**Figure 11:**
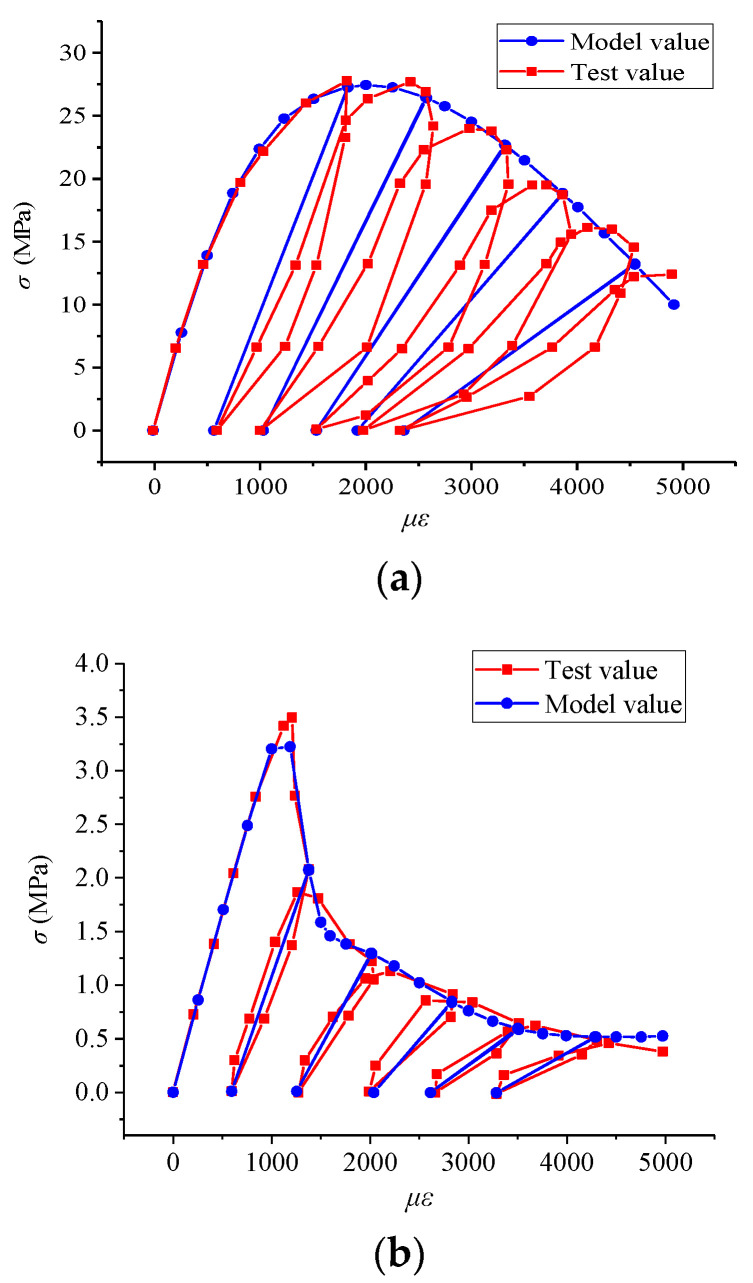
Mechanical behavior of concrete under cyclic loading. (**a**) Cyclic compression; (**b**) cyclic tension.

Further model validation was carried out based on the experimental results of uniaxial cyclic compression [29] and uniaxial cyclic tension [30], as shown in Figure 11. Compared with the experimental results, it can be seen that the model values are in good agreement with the test values.

### 4.2. Concrete under Biaxial Compression

**Figure 12:**
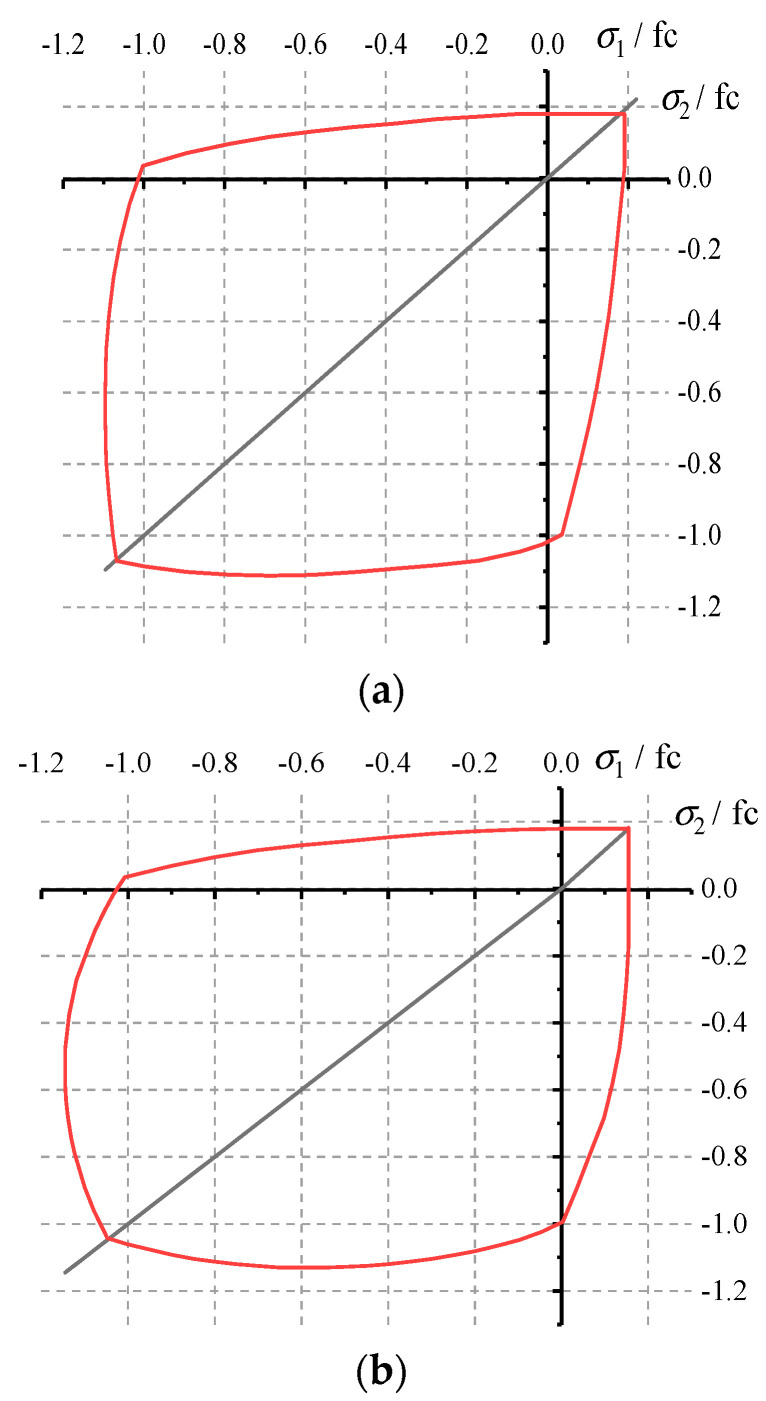
Comparison of model value and test value of concrete biaxial strength envelope. (**a**) Model value; (**b**) experimental value.

By substituting the identified random field parameters into the concrete biaxial constitutive equation in the above summary, the concrete biaxial strength envelope predicted by this model can be obtained. Figure 12 is a comparison diagram of model prediction results and test results [12] (Kupfer et al., 1969). It can be seen from the figure that the two are in good agreement. Therefore, it can be preliminarily verified that the model proposed in this paper can be used for the response and damage analysis of structures under biaxial loads. 

**Figure 13:**
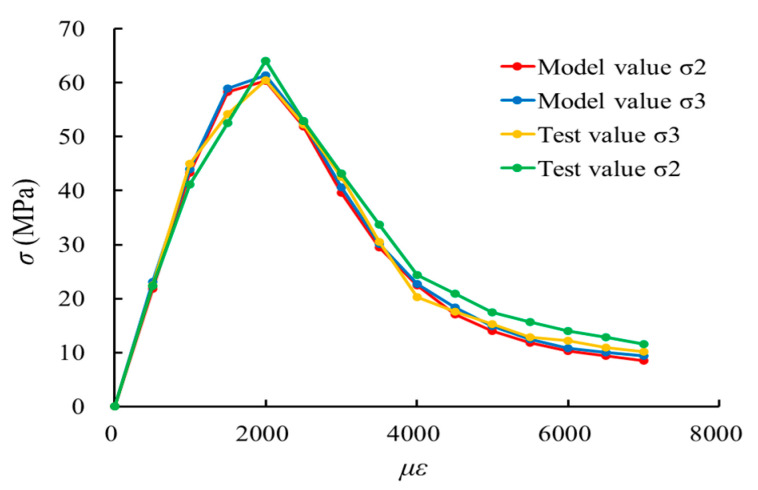
Simulation results of concrete under biaxial compression.

Further, according to the concrete biaxial compression tests ($\sigma_{3}=0,\sigma_{1}/\sigma_{2}=-1/-1$) conducted by Kupfer et al., the material parameters measured in the tests are elastic modulus *E* = 31,000 MPa and Poisson’s ratio *ν* = 0.2. Then, Figure 13 shows the comparison between the predicted biaxial stress–strain response of this model and the experimental results. The comparison results show that the predicted values of the model are in good agreement with the experimental results, which verifies the rationality of the biaxial damage constitutive model of concrete in this paper.

### 4.3. Analysis of Plain Concrete Structures

**Figure 14:**
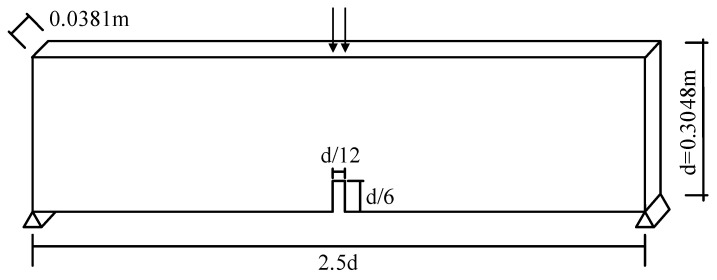
Schematic diagram of experimental model size and loading form.

The test of simply supported beams with notches in plain concrete under concentrated loads at the mid-span provides a good validation standard for the constitutive model of concrete materials [31]. The size and loading form of the test model are shown in Figure 14, and Table 2 shows the material parameters of the specimen.

**Figure 15:**
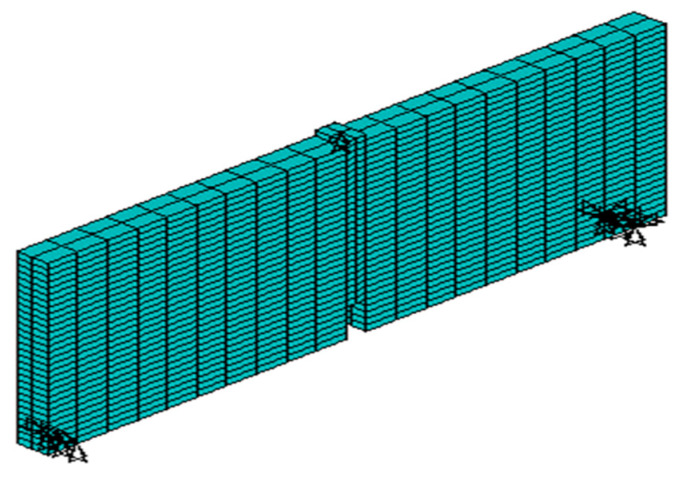
Finite element model.

**Figure 16:**
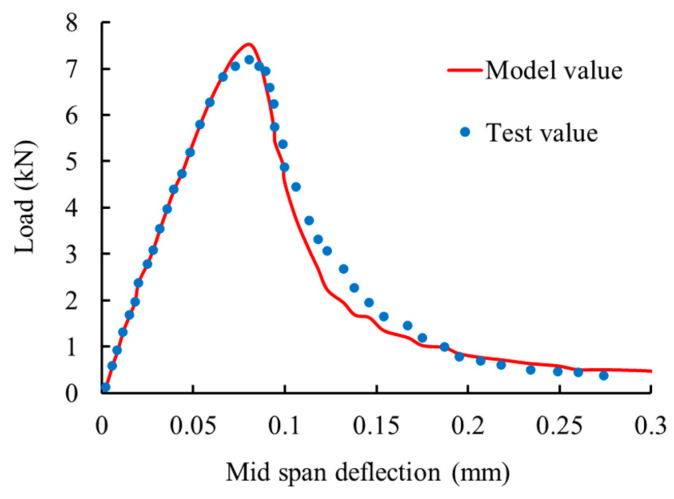
Load–mid-span deflection curve.

Then, establish a finite element model (Figure 15) using the constitutive model proposed in this article and calculate the load–deflection curve of a simply supported beam at mid-span, as shown in Figure 16. It can be seen that the experimental values are in good agreement with the model values, which verifies the feasibility of applying the model proposed in this paper to the calculation of concrete structures.

## 5. Conclusions

This article establishes a microscopic constitutive model with clear physical significance, which is composed of a spring element, plastic element, and friction element. The fracture of the spring unit represents the generation and expansion of internal cracks in concrete, the plastic unit represents the development of concrete plasticity, and the friction unit represents the mutual friction between crack surfaces. In addition, the random field theory is introduced into this model, which reveals the physical essence of the coupling of nonlinear and randomness of concrete. Then, the parameters of the random field are identified using the macro test data, and the multi-scale modeling of the concrete static damage constitutive model is realized. Finally, the model was developed as a subroutine to implement its application in finite element programs. The predicted values obtained from numerical simulation are in good agreement with the experimental values, which verify the rationality of the model.

## Figures and Tables

**Table 1:** Random field parameters identification results.

Parameters	$\lambda_{r}$	$\zeta_{r}$	$\lambda_{y}$	$\zeta_{y}$	Y	$\eta_{s}$	R
Compression constitutive	7.653	0.247	7.5	0.18	−0.316	0.077	1.019
Tensile constitutive	4.968	0.496	3.817	0.799	−0.08	0.06	0.613

**Table 2:** Material parameters of the specimen.

*E* (MPa)	*f_t_* (MPa)	*f_c_* (MPa)	ν	G_F_ (N/m)
27,413	2.886	34.0	0.18	40.29

## Data Availability

The data presented in this study are available on request from the corresponding author. The data are not publicly available due to privacy.
